# Deployable micro-traps to sequester motile bacteria

**DOI:** 10.1038/srep45897

**Published:** 2017-04-05

**Authors:** Raffaele Di Giacomo, Sebastian Krödel, Bruno Maresca, Patrizia Benzoni, Roberto Rusconi, Roman Stocker, Chiara Daraio

**Affiliations:** 1 Department of Mechanical and Process Engineering (D-MAVT), Swiss Federal Institute of Technology (ETH), Zurich, Switzerland; 2 Department of Pharmacy, Division of Biomedicine, University of Salerno, Fisciano, Italy; 3 Department of Bioscience, University of Milan, Milan, Italy; 4 Ralph M. Parsons Laboratory, Department of Civil and Environmental Engineering, Massachusetts Institute of Technology, Cambridge, MA, USA; 5 Institute of Environmental Engineering, Department of Civil, Environmental and Geomatic Engineering, Swiss Federal Institute of Technology (ETH), Zurich, Switzerland; 6 Department of Biomedical Sciences, Humanitas University, Milan, Italy; 7 Division of Engineering and Applied Science, California Institute of Technology, Pasadena, CA, USA

## Abstract

The development of strategies to reduce the load of unwanted bacteria is a fundamental challenge in industrial processing, environmental sciences and medical applications. Here, we report a new method to sequester motile bacteria from a liquid, based on passive, deployable micro-traps that confine bacteria using micro-funnels that open into trapping chambers. Even in low concentrations, micro-traps afford a 70% reduction in the amount of bacteria in a liquid sample, with a potential to reach >90% as shown by modelling improved geometries. This work introduces a new approach to contain the growth of bacteria without chemical means, an advantage of particular importance given the alarming growth of pan-drug-resistant bacteria.

Existing approaches to restrict the presence of bacteria in the environment are known to have important limitations. For example, antibacterial agents^1^,^2^ or antibiotics^3^ interfere with bacteria’s biochemical or genetic processes, but their excessive use poses serious concerns related to the emergence of pan-drug resistant strains^3^,^4^,^5^. Other methods use high-temperature^6^, or high-energy irradiation^7^. Pasteurization is an effective, widespread method^6^, but creates undesired free radicals and thermolytic byproducts. Gamma rays irradiation treatments^7^ require expensive equipment and a source of radiation. Microfiltration^8^ and sonication^9^ require considerable external energy. As these approaches are often invasive and expensive, the development of alternative or complementary strategies to reduce bacterial loads will be extremely beneficial in a broad field of applications.

Here, we propose to exploit the bacteria’s dynamic behavior to control the load of bacteria in liquid environments, by leveraging their motility and interaction with surfaces to trap them into microscale engineered particles. Key human pathogens, such as *Salmonella enterica, Helicobacter pylori, Vibrio cholerae, Vibrio vulnificus* and *Pseudomonas aeruginosa* and virulent strains of *Escherichia coli* are motile: they use their flagella to reach specific niches in the host and in several cases also as virulence factors. For example, *Salmonella* – which is found in contaminated food and water and represents the causative agent of gastroenteritis^10^ – swims towards and adheres to gastro-intestinal villi before entering the intestinal cells^11^,^12^,^13^,^14^. Enterobacteriaceae in general live in close association with surfaces^15^, which can generate attractive hydrodynamic forces on swimmers resulting in their accumulation in the proximity of boundaries^15^,^16^,^17^,^18^,^19^,^20^,^21^,^22^,^23^. These forces provide specific tropism to the target site on the intestinal cell surface, thus permitting the pre-docking phase at the onset of the infection^15^.

The understanding of how bacteria move and interact with surfaces has over the last decade been significantly furthered by the advances in microfabrication techniques^23^,^24^,^25^. For example, the effect of surfaces in redirecting bacteria has been exploited in the design of two-dimensional (2D) funnel walls. These structures favor bacteria crossing funnels in one direction over the other, based on the surface interaction of individual swimmers, and have been used to rectify the random swimming of bacteria and thereby concentrate them^26^,^27^,^28^,^29^,^30^. However, funnel walls have only been realized on surfaces, and have therefore not been deployable in liquid samples to date. Here, we leveraged recent advances in the resolution of 3D printing to develop three-dimensional (3D) micro-traps that rectify the motility of bacteria and trap them into deployable, microscale particles.

## Results

**Figure 1:**
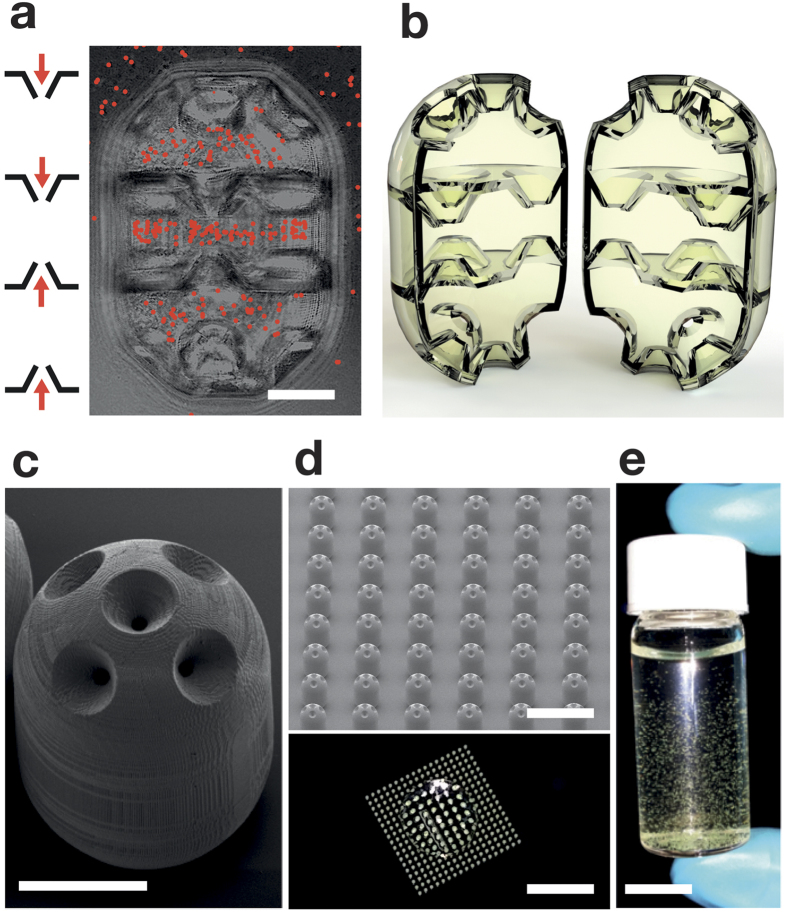
Micro-traps for motile bacteria. (**a**) Schematic of the rectifying funnel apertures and optical microscopy image of a micro-trap, scale bar 50 μm. Red dots: bacteria emphasized from a frame of the Supplementary Video 1. (**b**) Computer aided design 3D model of a micro-trap rendered in semi-transparent material, similar to the one used in the experiments, cut vertically into two halves to show the internal funnel structures. The inner volume of the structures calculated from this model is 1.72 nL. (**c**) SEM picture of a micro-trap. Scale bar, 80 μm. (**d**) 3D micro-traps on glass before detachment. Top panel: SEM image; lower panel: optical image. Scale bars, respectively, 500 μm and 5 mm. (**e**) Suspension of micro-traps in water. Scale bar, 1 cm.

We designed, fabricated and validated deployable micro-traps (150 μm in diameter and 220 μm in height) akin to miniaturized lobster pots that can sequester motile bacteria from a liquid suspension (Fig. 1a). Each micro-trap consists of chambers stacked to produce an egg-shaped particle (Fig. 1b,c). Funnel apertures (see close-up image in Supplementary Fig. S1) connect inner chambers of the micro-traps with the outside liquid. We printed arrays of 300 micro-traps on a glass substrate (Fig. 1d) using a 3D direct-laser-lithography system (Nanoscribe™; Methods and SI). After fabrication, the micro-traps were detached from their support substrate and deployed in a bacterial suspension (Fig. 1e).

**Figure 2:**
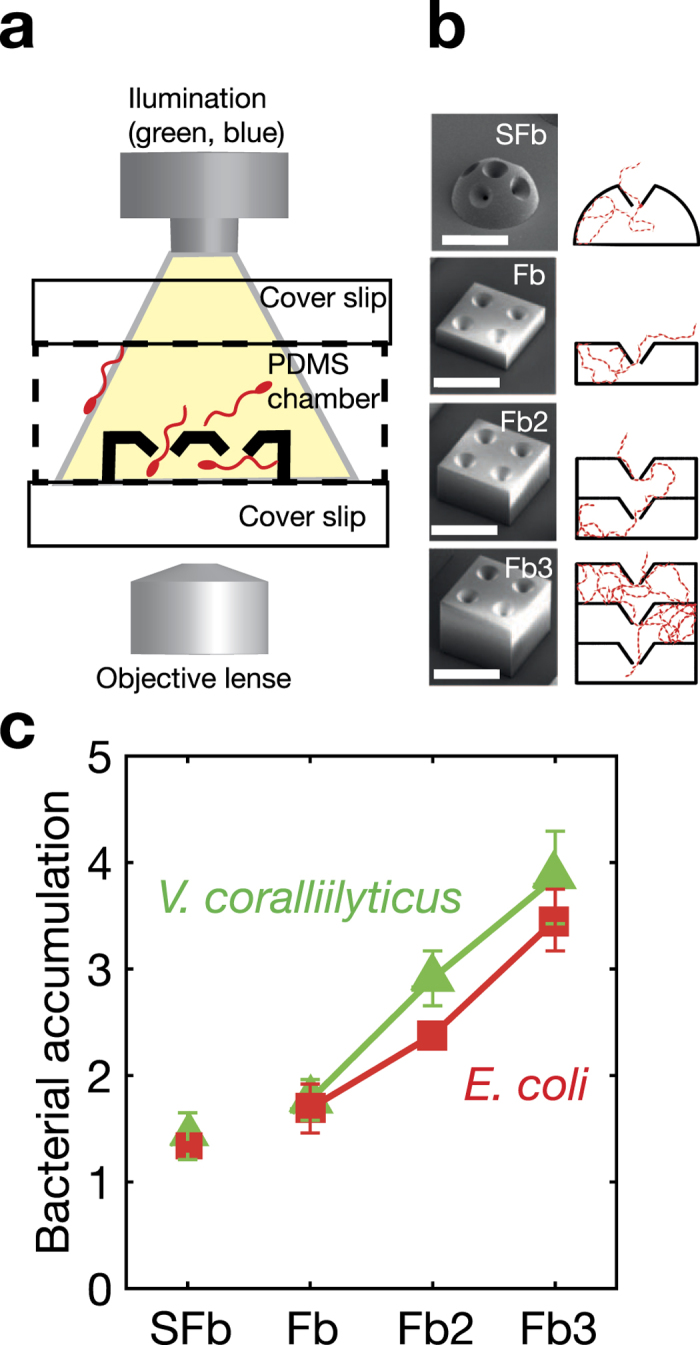
Accumulation capacity and trapping efficacy of surface-attached micro-traps. (**a**) Structures are attached on a cover glass with a PDMS gasket under an inverted fluorescent microscope. (**b**) SEM images (left-hand side) and cross-sections (right-hand side) of dome-shaped (SFb) and multilayer squared micro-structures (Fb1, Fb2, and Fb3). Scale bar, 100 μm. Squared micro structure having a size of 150 μm × 150 μm × 50 μm and 4 funnel apertures having an external diameter of 45 μm and an internal diameter of 10 μm (Fb). The height of the apertures in z-direction is 25 μm. Structures with 1, 2 (Fb2) and 3 (Fb3) stacked squared microstructure. The distance between two sequential apertures in z-direction is 25 μm. Dome-shaped micro structure (SFb) having a diameter of 150 μm and 5 funnel apertures with an external diameter of 45 μm and an internal diameter of 10 μm. The wall thickness of all structures is 8 μm. Red dashed lines: Simulated trajectories of a single bacterium swimming in different cross sections. (**c**) Bacterial accumulation for different surface-attached micro-traps and for two bacterial species after about 3 hours: *E. coli* (Red squares) *V. coralliilyticus* (green squares). Error bars correspond to the standard error of the mean for at least 3 independent experiments.

We first tested surface-attached micro-traps by imaging (Fig. 2a) the accumulation of bacteria within the traps over time, for four different geometries (single domes, 1-layer boxes, 2-layer boxes and 3-layer boxes; Fig. 2b), all with funnel-shaped apertures. We conducted experiments with two species of motile bacteria: the peritrichous enteric bacterium *Escherichia coli*, which represents the classic model for bacterial motility (i.e., run-and-tumble), and the marine pathogen *Vibrio coralliilyticus*, which possesses a single polar flagellum and swims rapidly with a strategy that significantly differs from *E. coli*’s (ref. 24). We quantified the concentration of bacteria inside the micro-traps by image analysis and compared this with the concentration of bacteria in the external suspension, determined with the same approach (Methods). In all micro-traps, we detected an accumulation of bacteria, for both *E. coli* and *V. coralliilyticus* (Fig. 2c), demonstrating the ability of the micro-traps to trap swimming bacteria. The highest average accumulation (4-fold higher concentration of bacteria within the micro-traps than outside) was observed in the 3-layer micro-traps. Furthermore, the accumulation increased with the number of layers in the micro-trap: the 3-layer traps had approximately double the accumulation compared to the 1-layer box (Fig. 2c), demonstrating that the multi-layer design is effective in enhancing trapping. The increase in accumulation with increasing number of layers is also in agreement with earlier experiments with 2D arrays of funnels^26^.

The trapping mechanism is based on the interaction of motile bacteria with 3D funnel-like apertures and relies on rectification and confinement. Upon approaching the micro-traps by random motility, bacteria preferentially swim along the surface of the funnels and this – due to the funnel’s shape – directs them through the funnel’s aperture and into the inner chamber. The asymmetric shape of the funnels then makes the likelihood that a bacterium swims out smaller than the likelihood that it swims in, resulting in an accumulation of cells in the micro-trap. This effect is enhanced by the presence of multiple layers of funnels, which guide bacteria further into the interior of the micro-traps and decreases the outward flux of bacteria.

The influence of micro-trap geometry on the trapping efficiency was further investigated through a mathematical model – previously validated in the presence of flow^31^ – which describes the spatial distribution of swimming bacteria, including the steric effect of the boundary on the cell swimming direction in the proximity of the surface (Methods). We simulated 10^5^ bacterial trajectories for each geometry and quantified the percentage of trapped bacteria at its steady state (the simulated trajectories are displayed in Fig. 2b and the steady state accumulation is displayed in Supplementary Fig. S3). We found that funnel-like apertures accumulate 35% more bacteria than cylindrical apertures (Supplementary Fig. S3). Cylindrical apertures that form an acute angle with the micro-traps’ internal walls, also trap bacteria due to their asymmetry toward the inside of the micro-traps. The predicted accumulation increased up to approximately 3-fold for the 3-layer micro-trap (Supplementary Fig. S3). These results compare well with the experimental observations and confirm that the mechanism of bacterial accumulation in the micro-traps is the rectification of bacterial motility due to the interaction with solid boundaries., a fundamental process likely applicable to all motile bacteria. This gives confidence that this approach of sequestering bacteria is feasible for a wide range of microbial swimmers, notwithstanding the need for additional species-specific studies.

**Figure 3:**
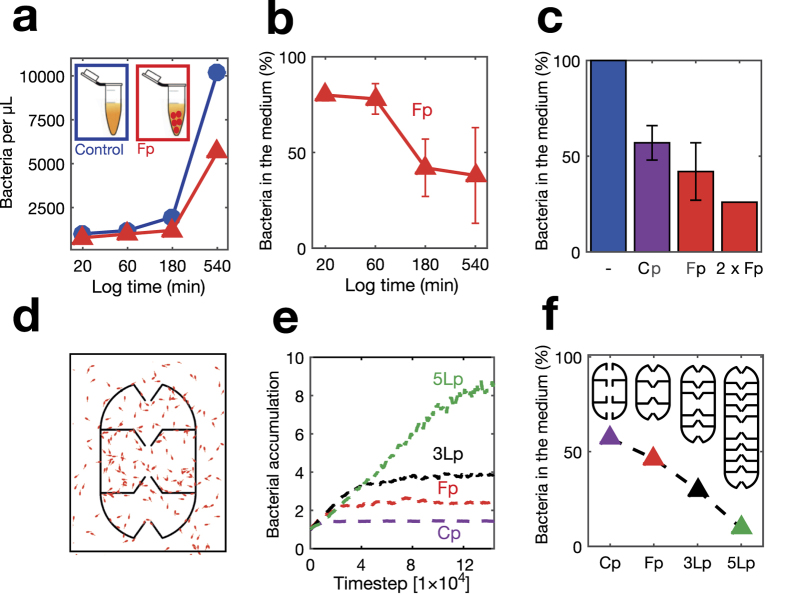
Bacterial depletion with deployable micro-traps, from experiments (**a**–**c**) and numerical simulations (**d**–**f**). (**a**) Red triangles: number of *E. coli* bacteria per μL present in a tube with 30 micro-traps (Fp) per μL as a function of time. Blue dots: control experiment without micro-traps. (**b**) Percentage of bacteria left in the suspending medium as a function of time, in the presence of 30 micro-traps (Fp) per μL. Error bars correspond to two independent experiments. (**c**) Percentage of bacteria left in the suspending medium after 180 minutes. Blue bar (-): tube without micro-traps. Violet bar (Cp): tube with micro-traps having straight apertures, concentration 30 per μL (4 independent experiments). First red bar (Fp): tube with micro-traps having funnel apertures, concentration 30 per μL (5 independent experiments). Second red bar (2 × Fp): tube with micro-traps having funnel apertures, concentration 60 per μL. (**d**) Simulated bacterial distribution in the case of a micro-trap with funnel apertures and 2 layers (Fp). (**e**) Time course of the accumulation obtained from numerical simulations of micro-traps with 2 internal layers (Cp, Fp), 3 internal layers (3Lp), and 5 internal layers (5Lp). (**f**) Maximum depletion reached for simulated micro-traps with 2 internal layers (Cp, Fp), 3 internal layers (3Lp), and 5 internal layers (5Lp).

We next deployed micro-traps in a bacterial suspension to test their ability to lower the bacterial load. The deployable micro-traps were designed by stacking a single dome (Fig. 2b) on top of a 1-layer box (Fig. 2b) and mirroring this stack to obtain an egg-shaped particle (Fig. 1b). Thus the deployable structures are a combination of the building blocks tested in Fig. 2b. We added 300 micro-traps to a 10 μL suspension of *E. coli* (Methods). The traps’ total internal volume was 5% of the total suspension and the volume displaced by the micro-traps was approximately 2% (Methods). Counting of bacteria revealed that the micro-traps progressively lowered the bacterial load in the suspension compared to the load in a simultaneous control, resulting in a 20% decrease after 20 min and a 60% decrease after 180 min (Fig. 3a,b). After approximately 180 min, the depletion of bacteria almost plateaued, owing to the competition between the flux of bacteria into the trap due to swimming rectification and the flux of bacteria out of the trap due to random motility. After 540 min the reduction in bacterial load compared to samples not containing the micro-traps was ca. 70%, while the absolute concentration of bacteria was 4 times higher compared to that measured at 180 min, due to bacterial growth. The constant or higher depletion of bacteria regardless of their growth in number proves that trapping is a phenomenon independent from the absolute number of bacteria per unit volume in the 10^3^–10^5^ bacteria/μL range (Fig. 3a). Importantly, the bacterial depletion was due to trapping within the micro-traps, as we did not observe a significant number of bacteria adhered to the traps’ outer surface (see Supplementary Video 1).

The depletion efficiency was affected by both funnel geometry and number of micro-trap. Comparing the bacterial depletion (measured after 180 min and normalized by the no-trap control) effected by 300 micro-traps with either asymmetric cylindrical apertures (images and design of the cylindrical-aperture micro-traps are shown in Supplementary Fig. S4) or funnel-like apertures showed that the latter were 22% more effective in depleting bacteria from the solution (Fig. 3c). This result indicates that funnel geometry is an important design factor that can be optimized, possibly in a species-specific manner, to achieve highest trapping efficiency in different applications. Doubling the number of micro-traps, from 300 to 600, resulted in a 15% increase in the bacterial depletion after 180 min, from 60% to 75% (Fig. 3c). This finding is in line with the theoretical limit obtained considering micro-traps act independently: given that 300 micro-traps captured 60% of the bacteria, the additional 300 micro-traps were expected to capture 60% of the remaining 40% of bacteria, i.e. an additional 24% (still independent of the absolute number of bacteria). We also estimated the accumulation of bacteria *c*_*mt*_ inside the micro-traps imposing the concentration of bacteria in the absence of micro-traps (control experiments) to be 1 everywhere in the volume. When introducing the micro-traps the concentration inside them increases, consequently the concentration in the outside medium will be less than 1. We describe the higher concentration in the micro-traps as 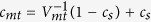 where *V*_*mt*_ is the internal volume fraction of the micro-traps with respect to the total volume and *c*_*s*_ is the concentration of bacteria in the suspension outside the micro-traps. This calculation revealed a 18-fold accumulation of bacteria (at time 180 min) within the micro-traps with respect to the concentration in the suspension. This value is larger than that measured in surface-immobilized micro-traps (Fig. 2c), probably due to the free motion of the deployed micro-traps in the suspension.

We performed 2D simulations of the deployable micro-traps to assess the effect of geometry on the depletion efficiency (Fig. 3d-f, Supplementary Fig. S5). In agreement with experiments, simulations showed that (i) the accumulation of bacteria in the micro-traps increases with time up to a plateau (Fig. 3e and (ii)) the accumulation was ~22% higher for micro-traps with funnel apertures compared to cylindrical apertures (Fig. 3f). Absolute concentrations of trapped bacteria were higher in simulations than in experiments, likely due to the 2D nature of the simulations.

In addition, the numerical model allowed us to predict the effect of more advanced trapping geometries, that vary the most influential geometrical parameter: the number of layers of the micro-traps. Such structures might be realizable in the future, considering further advancements in 3D printing technologies. The plateau depletion values obtained from simulations (Fig. 3f) have been normalized by the experimental depletion values of the cylindrical aperture micro-traps (Fig. 3c). We compared the bacterial accumulation in micro-traps with one, three and five internal layers and different aperture geometries (Fig. 3e,f; Supplementary Fig. S5). We found that the depletion of bacteria in the solution increases with the number of layers, from 60% for 1 layer to 75% for 3 layers and 95% for 5 layers (Fig. 3f). This is because, with more layers, bacteria are ‘stashed away’ further into the micro-trap and the flux of bacteria out of the micro-trap by random motility decreases. While the precise numbers will be different in 3D compared to 2D, we expect that the increase of the number of layers will also contribute very significantly to the efficiency of 3D micro-traps, and that the systematic optimization of the traps’ geometry will lead to yet more effective and faster accumulation. As the resolution of 3D printing improves, so will the possibilities for engineered microstructures that interact with microorganisms in controllable and potentially beneficial ways.

## Discussion

Our results demonstrate the potential of deployable micro-traps, which can be fabricated in high-throughput and can considerably reduce the load of bacteria from a liquid suspension within tens of minutes. This approach uses a completely passive mechanism that does not require heating, chemical additions or large amounts of energy. The intrinsic selection process favoring the trapping of the most motile (hence, often, most virulent) bacteria is a considerable advantage of this method. Our work also shows that the design of these structures can be guided by the extensive recent research focused on understanding microbial swimming and the interaction between microorganisms and surfaces, enabling the optimization of deployable microstructures and making their design species- and application-specific. This approach, in combination with continuing improvements in 3D micro-manufacturing, has the potential to reduce the number of micro-traps required to achieve the desired reduction in bacterial load by optimizing multiple elements of micro-trap design, including funnel geometry and number of layers.

Micro-traps can represent, in specific contexts, an appealing alternative to the use of pharmacological agents, such as antibiotics, whose extensive use has created a well-known red-queen effect by driving the emergence of resistant strains. In other applications, micro-traps may be used in synergy with antibiotics. For example, micro-traps could be loaded with antibiotics at resulting concentrations much lower than usually given in bulk – as the killing action will be localized inside the particles – and noxious effects of the antibiotics on the host are avoided. In this approach, rather than dosing antibiotics homogeneously everywhere, bacteria would swim into antibiotic-laden traps, and these could be further made more effective as well as potentially species-specific by augmenting the antibiotic with a chemo-attractants. These loading approaches will require the use of low-diffusivity compounds, or compounds partly trapped into a solid or gel matrix, to avoid diffusion severely limiting the time scale of micro-trap operation. Furthermore, after use, the micro-traps can be easily removed from the liquid using large-pore filters (e.g., pore size of ~100 μm), a cheap and fast filtering procedure. We envision this principle to be used to build micro-traps as diagnostic tools, possibly of much smaller size in the future, for deployment in the gut of animals and patients, for example to sample resident bacteria.

In summary, we have shown how 3D micro-technology can be utilized to reduce bacterial loads in a suspension, opening the road to a new “pharmacology” not based on chemistry, but on the possibility of interfering mechanically with the dynamic properties of pathogens.

## Methods

### Bacterial strains and growth conditions

*Vibrio coralliilyticus* strain YB2 dsRed, grown in Marine Broth 2216, and *Escherichia coli* strain AW405, grown in Luria Broth (LB) medium, were used in the experiment of Fig. 2. *E. coli* strain JM109, grown in LB medium, was used in the experiment of Fig. 3.

### Fabrication of micro-traps

To fabricate the micro-traps on the substrates and the deployable micro-traps, we used a semitransparent, negative tone photoresist (IP-Dip™) as the building material. The polymerized resist is biocompatible and has a low density and a Young’s module of 5 GPa (see SI Methods). The deployable micro-traps of Fig. 1a,c–e were 150 × 220 μm in size and had funnels with 45 μm and 10 μm diameter apertures, over a total length of 25 μm. To remove them from the substrate, we casted 20 μL of MilliQ^®^ water on top of the produced arrays (see Fig. 1d) and gently scratched the micro-traps with a sterile steel inoculation loop (Sigma Aldrich), allowing them to float. The micro-traps were then collected and freed of possible production residues by washing them in 0.2 mL tubes (Thermo-scientific), containing 100 μL of ultrapure water. The water-filled tubes with micro-traps were exposed to 50 mBar vacuum for 5 minutes and then spun for few seconds. The procedure was repeated until all micro-traps precipitated. We collected the supernatant, and the micro-traps were dehydrated under vacuum (50 mBar) for 1 h. The micro-traps were sterilized by exposing them to UV light for 30 minutes.

### Experiments on bacterial accumulation and depletion

For the experiments on bacterial accumulation with dome-shaped and multiple-layer box-shaped micro-traps (as shown in Fig. 2), we built a polydimethylsiloxane (PDMS) gasket around the bottom coverslip with the micro-traps attached. We added 50 μL of a bacterial suspension, and placed the samples in the testing apparatus. Since the volume of the bacterial suspension was much larger than the inner volume of each micro-trap, we could quantify the accumulation of bacteria inside individual micro-traps while neglecting the depletion of bacteria in the outer medium, *i.e.,* the micro-traps are considered immersed in an infinite bacterial suspension. To measure the accumulation of bacteria inside the microtraps, we counted the bacteria in a volume corresponding to an area of 40 × 40 μm and a height of 10 to 40 μm. We compared the obtained value with the number of bacteria in the same volume outside the structures at the same height from the bottom coverslip.

For the experiments on bacterial depletion with deployable micro-traps (shown in Fig. 3a–c), nine μL of LB medium were added to the tubes containing the micro-traps. To make sure that the medium had penetrated inside the micro-traps, a 50 mBar vacuum was applied again for 5 minutes. Control samples followed the same procedure. In each tube, we inoculated 1 μL of *E. coli*, grown in 5 mL of LB at 37 °C up to a density of ~0.7 OD_600_. The tubes containing bacteria and micro-traps were mounted parallel to the surface on a vertically rotating wheel at ~1.4 rpm at room temperature to avoid precipitation. At different time points (depending on the experiment), 2 μL of the supernatant from the respective samples were collected and the bacteria counted by optical microscopy (Olympus^®^ CKX41) in a Leja™ micro-chamber slide. Five different areas of the micro-chamber for each sample were photographed and analyzed by ImageJ software to determine the number of bacteria present. The counting method was validated with a separate experiment where OD reading were used as a reference (see Supplementary Fig. S6), the correlation between the two measurement was linear with R^2^ = 0.93.

For the time course experiments shown in Fig. 3a,b, we used independent sets of micro-traps for each time point, and the corresponding control samples. Starting from a single culture of *E. coli*, bacteria were divided into 8 Eppendorf tubes, 4 containing Luria Broth (LB) medium and 300 micro-traps each and 4 containing only LB medium. For the experiment of Fig. 3c each experiment was performed (together with its control) starting from an independent culture.

### Numerical simulations

A Langevin model of bacterial motility has been employed (see ref. 31), in which we modelled bacteria as prolate ellipsoids with aspect ratio q = 8.5, accounting for the combined hydrodynamic resistance of the cell body and flagellar bundle in *E. coli* (ref. 19), and swimming speed directed along the long axis. The equations of motion were integrated numerically for 10^5^ simulated bacteria using a fourth-order Runge-Kutta scheme implemented in Matlab© (The Mathworks^®^). An integration time step of 50 ms was used and simulations were run for up to 1.44 × 10^5^ time steps which corresponds to 2 hours of real time. The swimming speed was set to 15 μm/s and the rotational diffusivity – which takes into account random fluctuations in the cells’ orientation due to tumbling – to 0.4 s^−1^, typical values for *E. coli* (ref. 17). To model the interaction between the bacteria and the surface of the micro-traps, when a bacterium arrives within 1 μm from a surface as it moves towards the surface, we constrained its incident angle to be 2.5 degrees relative to the surface for one time step, while its swimming direction randomly changes due to rotational diffusion. The prescribed incident angle of 2.5 degrees was previously found to be the most stable angle for *E. coli* swimming near a surface^22^. We confirmed that variations of the incident angle in the range of 0 to 5 degrees had no qualitative effects on the simulations. Furthermore, the numerical model was validated for the case of bacteria swimming between two parallel surfaces (Supplementary Fig. S2), by comparing the predicted accumulation of bacteria near the surfaces with prior observations for *E. coli* (ref. 17). For further information on the numerical simulation, micro-traps’ design and realization, see Supplementary Methods.

## Additional Information

**How to cite this article**: Di Giacomo, R. *et al*. Deployable micro-traps to sequester motile bacteria. *Sci. Rep.* **7**, 45897; doi: 10.1038/srep45897 (2017).

**Publisher's note:** Springer Nature remains neutral with regard to jurisdictional claims in published maps and institutional affiliations.

## Supplementary Material

Supplementary Video 1

Supplementary Materials
