# Supplementary material for: Deployable micro-traps to sequester motile bacteria
Source: Sci Rep. 2017 Apr 5;7:45897. doi: 10.1038/srep45897 (PMC5381207; doi:10.1038/srep45897)
Supplement: Supplementary Materials [file srep45897-s2.doc]

**Supplementary Materials:**

**Deployable micro-traps to sequester motile bacteria**

**Raffaele Di Giacomo^1*^, Sebastian Krödel^1*^, Bruno Maresca^2^, Patrizia Benzoni^3^,**

**Roberto Rusconi^4,5^, Roman Stocker^4,5^, Chiara Daraio^1,6^**

^1^ Department of Mechanical and Process Engineering (D-MAVT), Swiss Federal Institute of Technology (ETH), Zurich, Switzerland.

^2^ Department of Pharmacy, Division of Biomedicine, University of Salerno, Fisciano, Italy.

^3^ Department of Bioscience, University of Milan, Milan, Italy.

^4^ Ralph M. Parsons Laboratory, Department of Civil and Environmental Engineering, Massachusetts Institute of Technology, Cambridge, MA, USA.

^5^Institute of Environmental Engineering, Department of Civil, Environmental and Geomatic Engineering. Swiss Federal Institute of Technology (ETH), Zurich, Switzerland.

^6^ Division of Engineering and Applied Science, California Institute of Technology, Pasadena, CA, USA.

*These authors contributed equally to this work

**SI Methods**

**Design and realization of the traps.** The projected geometries were exported as “.stl” files. Deployable micro-traps and boxes were fabricated using a commercial 3D direct-laser-lithography system (Nanoscribe™). In order to inform the machine with the geometry of the structure, the “.stl” files were converted into an input file by means of the commercial Describe™ software. We performed slicing of the geometry into separate layers of 0.55 μm thickness in the z-direction. Each layer was hatched into a number of lines with a distance of 0.35 μm. We used a different discretization in the x-y plane compared to the x-z plane due to the fact that the voxel had an elliptical shape and thus a different resolution in the two planes. A drop of a negative tone photoresist (IP-Dip™) was casted on top of a fused silica substrate (25 mm × 25 mm × 0.7 mm). The substrates were placed on a holder and inserted into the slot of the Nanoscribe™ machine. We used a 25x objective (LCI Plan-Neofluar), which was brought in direct contact with the resist (Dip-In Lithography). This writing setup enabled us to fabricate structures with heights up to 250 μm. The lines were scanned using the Galvo scanning mode of the Nanoscribe™ machine. The writing speeds were up to 45,000 μm/s. The fabrication of one deployable micro-trap took approximately 90 s. We fabricated arrays of 324 (18×18) deployable micro-traps with an overall runtime of 8 h. Arrays were allowed to develop by immersion into commercially available developer (mr-Dev 600™) for 35 min and subsequently rinsed in IPA for 10 min. Structures were very stable and hard to damage. The polymerized resist had a bulk density of ≈1,200 kg/m^3^.

**Supplementary Video S1**

The video shows a representative deployable micro-trap after 3 h of incubation, containing trapped bacteria moving inside it. The movie was captured with an optical microscope (OLYMPUS CKX41) equipped with a camera (OLYMPUS SC50) and displayed in real time.

**Funnel apertures**

Fig. S1 shows the funnel aperture used. The angle and length of the funnel walls were reproduced from the 2D case reported in Ref. 1.


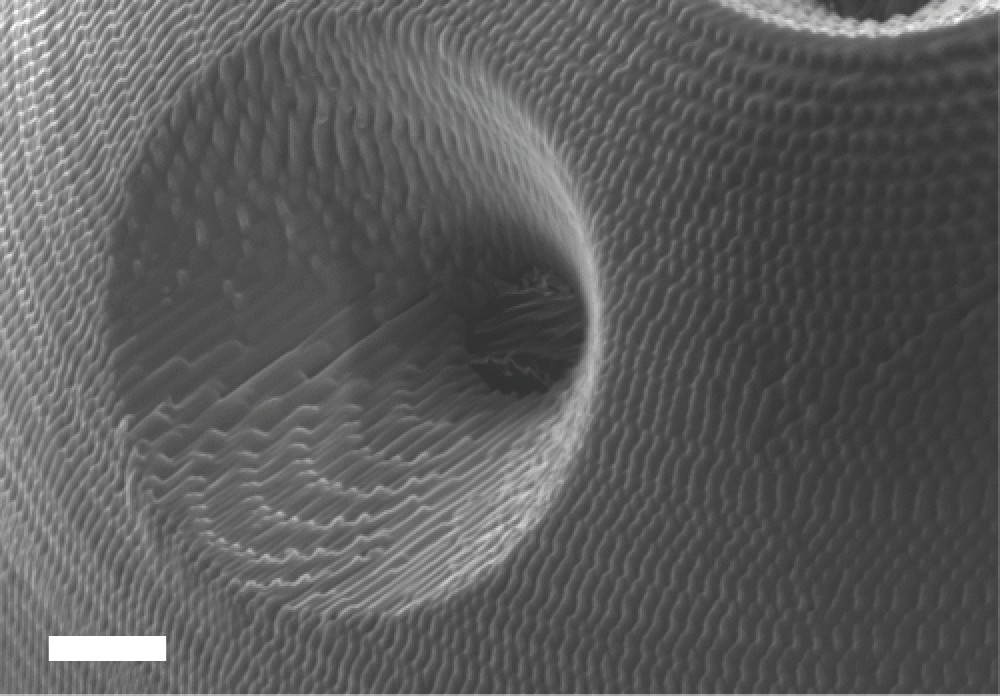


**Fig. S1 | Detail of the funnel apertures of the micro-traps.** Scale bar = 10 μm. The external diameter is 45 μm and the internal diameter is 10 μm. The depth of the aperture (from external to internal diameter) is 25 μm.

**Numerical model**

To validate the numerical model, we simulated bacteria swimming between two parallel surfaces (Supplementary Fig. S2), and compared the results to prior observations for *E. coli* (Ref. 2).

**Fig. S2 |** **Numerical results of bacterial accumulation between two parallel surfaces,** located 200 µm apart. Concentration of bacteria as a function of distance from the bottom surface.Inset: As a comparison, experimental results of *E. coli* accumulation between two parallel plates (Modified from ref 17).

**Surface-bound micro-traps simulation**

We simulated the trapping capabilities of the surface-bound micro-traps (Fig. S3). We find that, in accordance with experimental results, increasing the number of layers increases both the maximum accumulation in the innermost chamber and the percentage of trapped bacteria. Moreover, the simulated result shows that the trapping is not linearly dependent on the trapping volume. A linear increase in trapping volume from 1 to 3 layers results in a more than linear increase in the number of trapped bacteria.

**
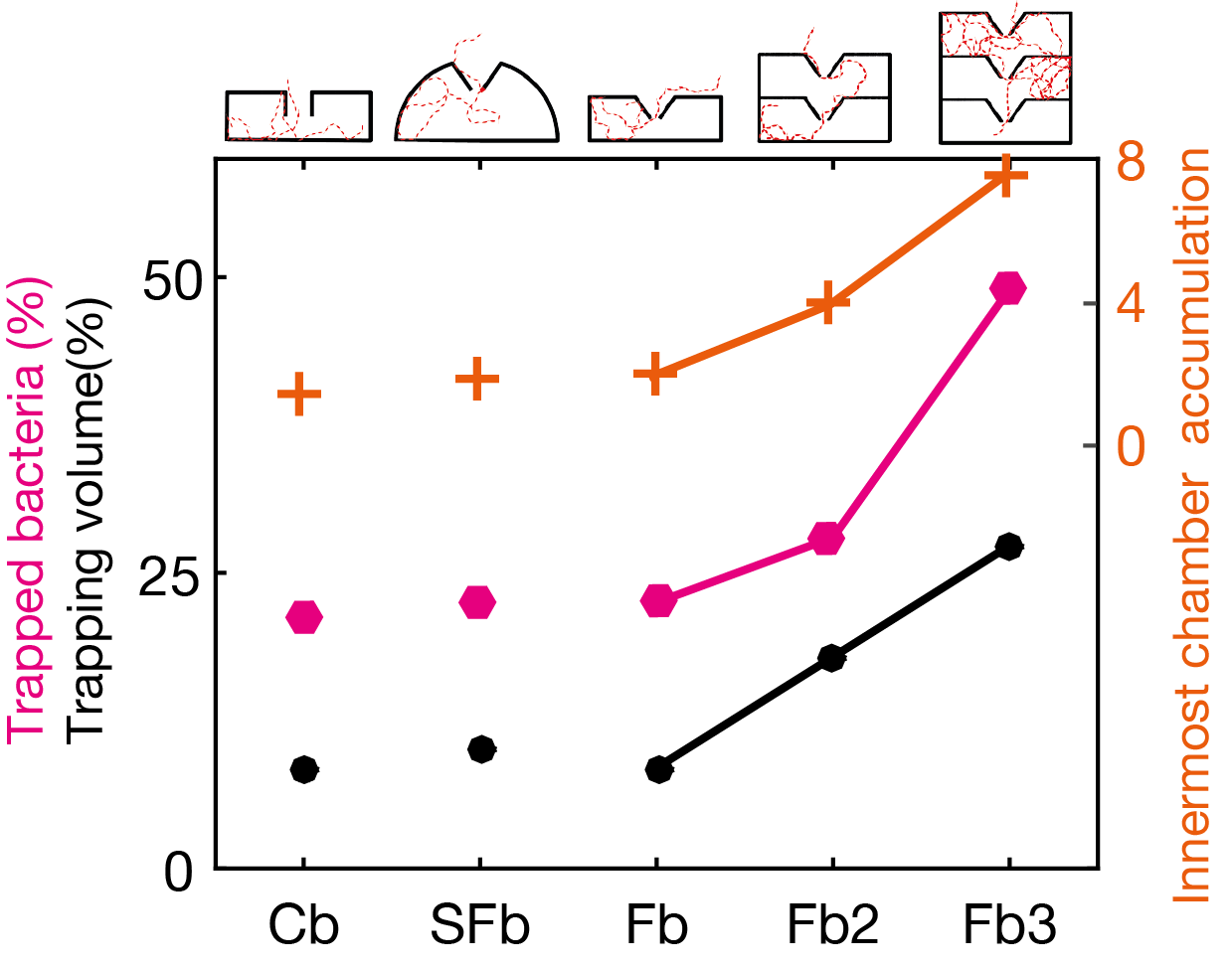
**

Fig. S3 | Simulated trapping performance of surface-bound micro-traps. Trapped bacteria (pink) and innermost chamber accumulation (orange) as a function of the trap geometry. Also plotted is the trapping volume fraction.

**Cylindrical-aperture deployable micro-traps**

Fig. S4a shows a deployable micro-trap with cylindrical apertures. A computer rendering in transparent plastic material is shown in Fig. S4b next to an optical microscopy image of a realized micro-trap in Fig. S4c. An SEM picture is also shown in Fig. S4d and a detail of the cylindrical aperture in Fig. S4e.

**
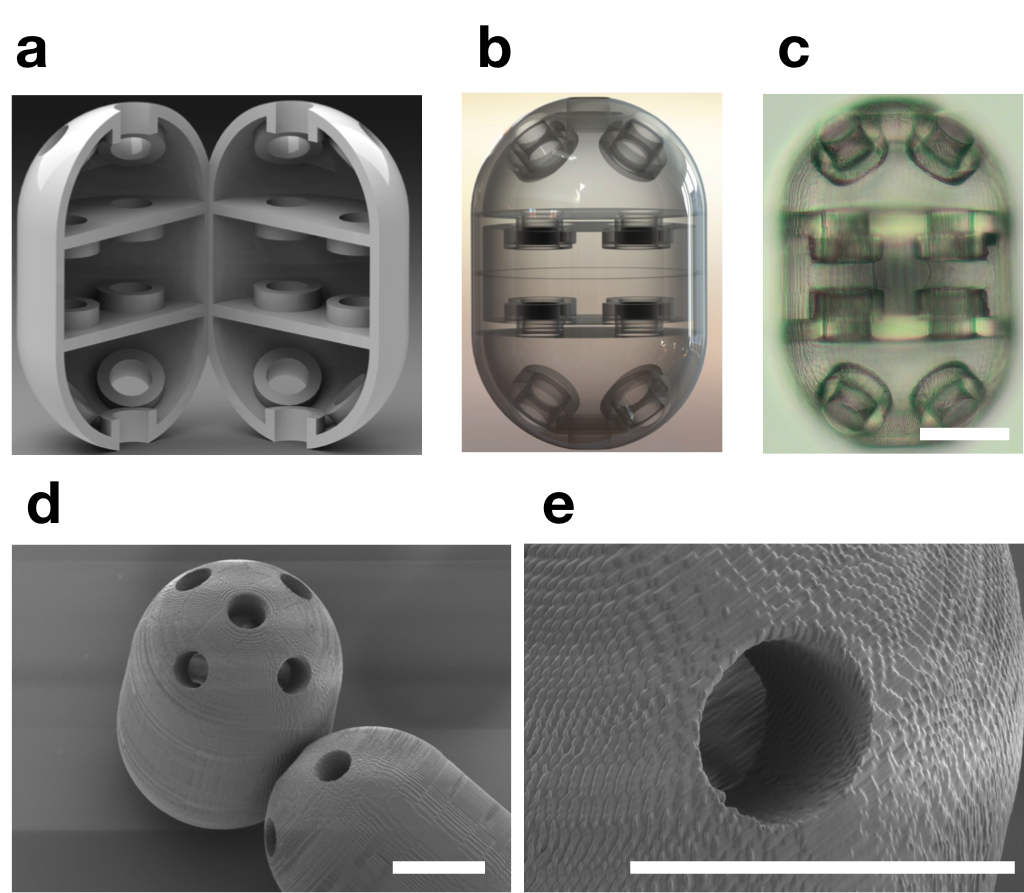
**

**Fig. S4 | Deployable micro-trap with cylindrical apertures. (a**) CAD 3D model cut vertically into two halves. The inner volume of the structures calculated from this CAD model was 1.64 nL. (**b**) CAD rendering of a micro-trap. (**c**) Optical microscopy image of a micro-trap (20x) (scale bar = 50 µm). (**d**) SEM picture of two micro-traps (scale bar = 50 µm). (**e**) SEM detail of the funnel geometry (scale bar = 50 µm).

**Accumulation in the deployable micro-traps**

Fig. S5 shows numerical simulations of 2D cross-sections of deployable micro-traps with 2, 3 and 5 layers, respectively. As reported in experiments shown in Fig. 3c, the micro-traps with funnel-shaped apertures show a higher accumulation in the inner layers compared to the micro-traps with cylindrical apertures (see Fig. S4). By increasing the number of layers a further improvement of the accumulation is achieved (see Figs. S5c,d and 3f).


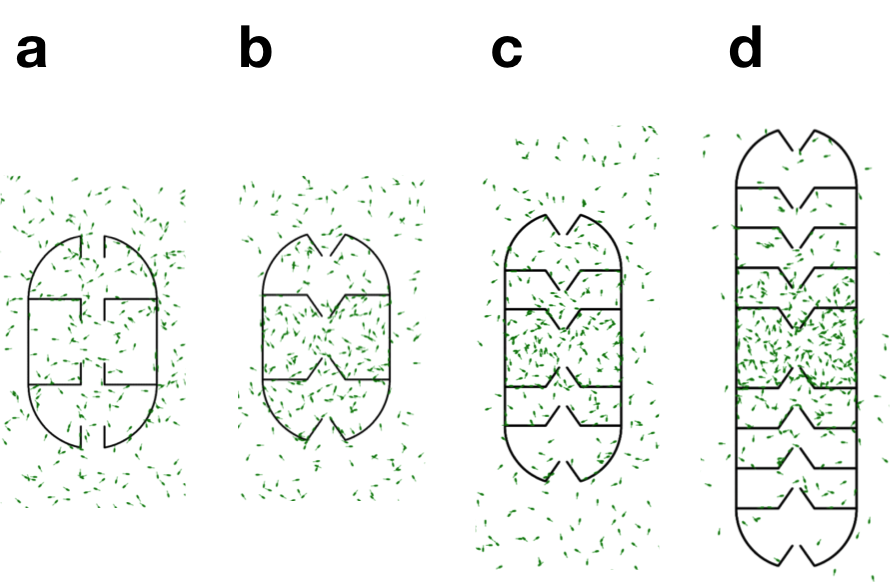


**Fig. S5 | Numerical simulations of the bacterial distribution.** (**a**) Bacterial distribution after 3000 s in the case of a micro-trap with cylindrical apertures and 2 layers (Cp). (**b**) Bacterial distribution after 3000 s in the case of a micro-trap with funnel apertures and 2 layers (Fp). (**c**) Bacterial distribution after 3000 s in the case of a micro-trap with funnel apertures and 3 layers (**d**) Bacterial distribution after 6000 s in the case of a micro-trap with funnel apertures and 5 layers.

**Bacterial density measurements**

To validate the bacteria counting method using Leja™ micro-chambers we compared those counts with standard OD readings (Fig. S6). We found that there is a linear correlation between the counts obtained with the two methods.


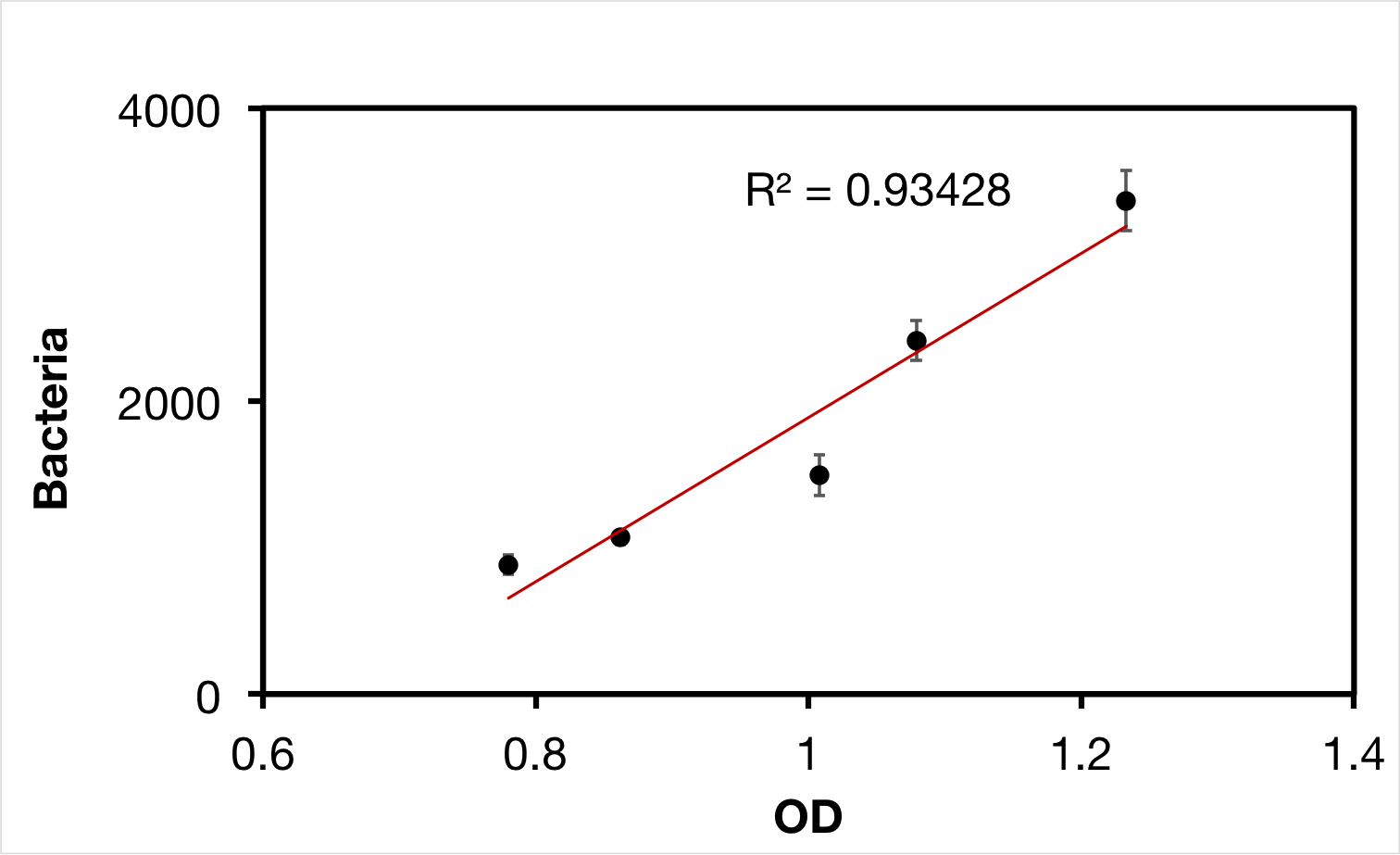


Fig. S6 | Bacterial density measurements. The number of bacteria obtained using Leja™ micro-chambers compared to the OD readings.

**References:**

1. Galajda, P., Keymer, J., Chaikin, P. & Austin, R. A wall of funnels concentrates swimming bacteria. *J. Bacteriol.* **189,** 8704–7 (2007).

2. Molaei, M., Barry, M., Stocker, R. & Sheng, J. Failed escape: Solid surfaces prevent tumbling of *Escherichia coli*. *Phys. Rev. Lett.* **113,** 68103 (2014)
